# Dropout, Autonomy and Reintegration in Spain: A Study of the Life of Young Women on Temporary Release

**DOI:** 10.3389/fpsyg.2020.01359

**Published:** 2020-06-30

**Authors:** Fanny T. Añaños, María del Mar García-Vita, Diego Galán-Casado, Rocío Raya-Miranda

**Affiliations:** ^1^Department of Pedagogy, Institute of Peace and Conflicts (IPAZ), University of Granada, Granada, Spain; ^2^Department of Education, University of Almería, Institute of Peace and Conflicts (IPAZ), University of Granada, Almería, Spain; ^3^Faculty of Education and Health, Camilo José Cela University, Madrid, Spain; ^4^Department of Statistics and Operations Research, University of Granada, Granada, Spain

**Keywords:** young people, school dropout, women, prison, autonomy, work, social reintegration, education

## Abstract

This study analyses the psycho-educational and social paths of women prisoners after the time they drop out of school as minors, based on different variables related to autonomy and their preparedness to face temporary release.

**Methods:**

Qualitative and quantitative methods were used to analyse a national sample of 310 women prisoners (30.1% of the population) in 31 prisons through a mixed-mode questionnaire and interview. We analysed the significant association of variables related to dropout and obtained a log-linear model that relates dropout to recidivism and Roma culture. Work experience was analysed using the McNemar test, and variables influencing the participant’s job at the time of the study were analysed by applying cluster analysis.

**Results:**

Young women comprise 66.6% of individuals who drop of the education system as minors (primary 49.3% and secondary 22%). They drop out between the ages of 7 and 17, and have traits of greater vulnerability than those who stayed in school until adulthood. In this population, we find a significant association with various factors: belonging to Roma culture, having family members in prison and delinquent recidivism; and higher unemployment (43.4%) and low income before entering prison. This situation is increasing today. In prison, these women had more connection to education/training, which can improve their employability. They also encounter difficulties with personal security, decision-making, personal/professional dependence, planning for the future, administrative matters and handling information and communication technologies, job-seeking skills, etc. Their self-perceived strengths are, however, assuming responsibility, taking orders, respecting schedules and timetables, working on a team and feeling prepared to start a job, as well as having optimistic convictions about the future.

**Conclusion:**

The vulnerabilities and risk factors studied have a negative influence primarily on processes of personal, social and job autonomy in female minors who left the education system. Yet these minors show factors of protection and resilience. On temporary release at the time of the study, they face the consequences that their prison terms and incarceration have for their perceptions, attitudes, competencies and future prospects, as well as social marginalization and stigma. Early, coherent socio-educational interventions are thus needed to improve social integration-reintegration.

## Introduction

The topic of education is sometimes questioned or provided in unfavourable conditions, depending on the contexts in which it occurs. Yet education is a human right (ONU, 1948) that nations are required to protect and enforce, independently of context. It should transcend the barrier of prison walls or environments of confinement (Añaños et al., 2019). In Spain, in fact, the goals of punishment that deprives people of freedom are oriented to social reintegration and re-education of convicts (Spanish Constitution, 1978; Ley Orgánica 1/, 1979).

The prison population in Spain is 58,917, of whom 92.55% are men and 7.45% women (Secretariat-General of Prisons [SGIP], 2019a). This figure is one of the highest in Europe, after the United Kingdom, France, Germany and Italy. The total world prison population is around 10.74 million, and the countries with the largest prison populations are the United States of America, China, Brazil, the Russian Federation, Thailand, Indonesia, Turkey, Iran, Mexico and the Philippines (Walmsley, 2019). Women prisoners number 714,000, constituting 6.9% of the global prison population (Walmsley, 2018). These figures reveal the tremendous importance of and need to understand educational careers prior to prison and the role of the penal institution in this matter.

This study analyses early school dropout, taking as reference the age at which the person dropped out, considering that education levels prior to higher education generally end at 18. At the same time, it analyses the different risk factors that influence the person’s situation and the impact of prison itself (all of which are related to personal, social and job autonomy for women on temporary release in Spain) and the influence of temporary release on social reintegration.

### Remaining in the System or Dropping Out: Factors That Influence Personal Autonomy

The current Spanish Education System is governed by the Organic Law for the Improvement of Educational Quality 2013 (Ley Orgánica 8/, 2013), which is divided into two parts, compulsory (6–16 years of age) and non-compulsory. Non-compulsory education generally includes pre-school (0–4 years of age), non-compulsory secondary education (university-preparatory and vocational training, 16–18 years of age), and subsequently university, as well as adult education, which is governed by its own law.

According to the (Ley Orgánica 8/, 2013), basic compulsory education includes the acquisition of a set of legally established competencies, and education systems in the European Union have strict legislation requiring students to attend this schooling (Martínez and Echeverriá, 2009; González Losada et al., 2015). This approach leads students to remain in school even when their performance is not necessarily associated with success (Morales Sequera and Pérez Sparrels, 2011). Merely remaining in school without academic achievement (failure) can become a risk factor for interrupting the educational itinerary of non-compulsory instruction (dropout).

Failing in school is defined as a situation in which the student does not achieve the goals generally established for the age group (Sarceda-Gorgoso et al., 2017). Failure can occur at different levels of compulsory education. It is the result of a long process (Janosz et al., 2013) or “continuum” that goes beyond the individual decision taken at a specific moment (Jimerson et al., 2000) and involves making mistakes, losing self-confidence, feeling discouraged, investing less effort and increasing the possibilities of failing again (Kamal and Bener, 2009). It may also involve disagreements with the school and its labelling dynamics (Mena Martínez et al., 2010).

The decision to drop out can be conditioned, among other factors, by personal circumstances and perspectives, which are often associated with loss of confidence or lack of motivation (Kamal and Bener, 2009), the influence of a family and social context that does not prioritize school (Melendro, 2008; Añaños-Bedriñana, 2013; García-Vita, 2017) or disagreements with the school itself and its dynamics (Cohen, 2006; Rue, 2006). Dropping out can be understood as an expression of autonomy in young people and adolescents, especially for women. At the same time, dropout affects young women’s development multidimensionally and in its different dimensions (Casanova, 2016).

Autonomy during adolescence has three dimensions (Noom et al., 1999; Parra et al., 2015): behavioural (capability to act independently), cognitive (acquisition of competencies) and emotional (self-concept, self-confidence, relationships with an egalitarian and symmetrical emotional foundation). Dropout is also intimately related to the meaning that the student gives to his/her experience (Crozier, 2001). Thus, the most disadvantaged groups perceive school as an environment that does not attend to their personal needs (Willis, 1988), generating a clear culture of resistance to prevailing legitimation (Escudero Muñoz, 2005). Repeated and prolonged negative experience leads to processes characterized by early dropout, and thus the impossibility of passing or obtaining a specific accrediting degree.

In the case of the most vulnerable populations, young people who have stayed in the education system (typically until 18 years of age) without success or who have dropped out and ended up in prison have a series of characteristics that put them at higher social risk (contexts with situations of family breakdown, conflict, violence, addiction, delinquency, prior incarceration, culture that does not bid for school, etc. (Añaños-Bedriñana, 2012, 2013; Añaños-Bedriñana et al., 2019; Añaños et al., 2019). These individuals face, if possible, even greater consequences – not only lack of educational competencies but also greater difficulties in obtaining personal and social development. These stages of development are, specifically (Parra et al., 2015), the capability to act independently and to take control of one’s own life, and the perception of independence through self-confidence.

This reality means having fewer opportunities for active participation in economic life, as well as precarious opportunities for work and less stability in each opportunity (Fortin et al., 2005). We can also infer that such individuals have not acquired the skills needed to participate in other vital phases of life, skills such as healthy leisure and involvement in civic and cultural affairs (Ferguson et al., 2005). Ultimately, the person in this situation has serious difficulties acquiring a series of rights that should be guaranteed through education (Bernstein, 1996).

All of these factors related to failing in school, remaining in the system without a degree or early dropout and its consequences also compromise autonomy and the processes needed to live a decent life in contemporary society. This study shows the differing paths of women who dropped out of the education system as minors and of women who remained in the system at least to the age of 18, all of whom are now serving a sentence in the Spanish prison system.

### Prison and Autonomy: Impact on the Life of the Person and Education Possibilities

The concept of autonomy has been developed extensively through different disciplinary approaches and in different contexts and populations, but few of this focus on the context of prison. Kantian autonomy involves self-governance and self-control according to principles one has chosen for oneself. This means that moral agents are subject to objective, rationally moral principles (Power et al., 1989; McDonough, 2005). Authors such as Piaget and Kohlberg, who ground their ideas in Kant, describe autonomy as the capability to create moral norms that we live by. This perspective is qualified by authors like Gilligan (1982), who introduce questions of attachment into the theory, structuring it around the category of gender and rejecting an individualistic focus. All of this theoretical corpus requires adjustments to make it relevant when studying an inmate population in a prison setting. As Parrón (2014) argues, autonomy is contextualized by and connected to different areas of life. Prison, however, assumes functions of attention, care and control of the life of its inhabitants, relativizing autonomy although not negating it.

The topic of autonomy in prison has also been approached from the perspective of the increase in studies of incarceration. Authors such as Goffman (1987) and Clemmer (1958) reveal the patent difficulty for inmates of exercising and developing autonomy on entering the prison or carceral subculture, where the question of the relationship between adaptation and preservation of identity and autonomy emerges. The less one fits or adapts to the prison culture, the greater one’s degree of autonomy (Goodstein, 1979).

Characteristics of a context of confinement are unified and institutionalized, in turn presenting a multiplicity of opportunities and diverse experiences. The prison sentence in Spain is oriented to achieving rehabilitation reintegration and re-education of people who serve the sentence (Art.25.2 Spanish Constitution, 1978; Ley Orgánica 1/, 1979; Real Decreto 190/, 1996). Achieving this goal involves understanding the process of the prison sentence – in the terms of the legislation it self – as an instrument of personal change that goes beyond mere punishment (Gil Cantero, 2010; Añaños-Bedriñana, 2013).

According to Toch (1975), from an ecological-systemic perspective, adjusting to the penitentiary environment is a dynamic transaction between person and environment. Autonomy is not understood as a mere individual process of decision making but is grounded in the relational (Ennuyer, 2002) and in the presence of interdependence among choices, skills and resources (Le Coadic, 2006). If we analyse this specific factor inside prisons, we run up against the constant need to obey orders and premises as measures for achieving proper organization of prison life and maintaining security (Hinojosa, 2009; Gallego et al., 2010). Subjects who live together in prison depend on the channels established by the institution itself, which leads to a process characterized by depersonalization and high degrees of stress and anxiety (Arroyo-Cobo and Ortega, 2009; Galán Casado, 2015). Further, autonomy should occur free of external pressures, such as group pressure, or internal pressure, such as feelings of guilt, shame and anxiety (Radel et al., 2013). These conditions are difficult to fulfil in an environment where human relationships are distorted, organized hierarchically, intensified and institutionalized (García-Vita and Melendro Estefanía, 2013).

In order to avoid this destabilizing effect, it is important to stress one of the main instruments for acquiring values, knowledge, competencies, skills and attitudes: education. Incarceration can be a turning point for some people (Johnson and Dobrzanska, 2005), an opportunity to increase pro-social attitudes and improve emotional well-being (Wormith, 1984; Zamble and Porporino, 1988; Zamble, 1992). The activities inherent in the prison routine – such as work, educational or sociocultural activities, and the relationships established with the prison staff – are positively related to feelings of autonomy and well-being of the male and female inmates (Goodstein et al., 1984; Gover et al., 2000; Liebling, 2004; García-Vita and Melendro Estefanía, 2013; Van der Laan and Eichelsheim, 2013).

Alone these lines, education in penal environments serves as a refuge (Ruess, 1997), a safe space (Szifris, 2017; Ruíz Cabello and López-Riba, 2019) that distances the inmate from other dynamics resulting from unemployment and the complex power relationships that dominate prison life (Clemmer, 1958); but above all education in prison is an educational time and place (Núñez, 2010). This situation is actually the first step toward preparing for life in freedom, since the mere fact of living for a long period of time in the prison lifestyle generates a series of routines that will not work in the environment outside prison and that become obstacles for optimal resocialization.

The educational model in Spanish prisons is organized around two complementary channels, a formal channel that corresponds to the official education system and a second channel involving treatment-based intervention in the prison.

The first group of options includes adult education. Inside the prison, inmates are provided basic education, divided into two stages. Level I is oriented to literacy and Level II to more specific and extensive content (García-Tardón, 2019). Compulsory secondary education (ESO), which constitutes the second stage of compulsory education (in this case, termed Compulsory Adult Education (ESA), is delivered in a mixed distance and classroom format. Subsequently, the inmate can pursue non-compulsory education, such as non-compulsory university-preparatory education, to obtain the degree that permits access to higher education; or Vocational Training (FP). At this middle level (secondary, not higher, education) students can only pursue mid-level educational cycles (of 2 years), and these are only available in a limited number of prisons. The last option is university or higher education, delivered by the Spanish National Distance Education University (UNED), through the specific modules imparted, each with its own organization and structure (Secretariat-General of Prisons [SGIP], 2019b).

The second group contains various types of so-called specific programmes – work-related, socio-educational, etc. – as part of the institution’s treatment-related intervention. These programmes are organized into four major sections (Secretariat-General of Prisons [SGIP], 2018): programs for cultural enrichment, libraries and the promotion of reading, recreational sports activities and competitive sports programmes. The activities are proposed by collaborating organizations, the inmates and the prison itself. Both formal education and the treatment-based activities form part of the Personalized Treatment Plan (PIT) that each inmate develops. The inmate’s development and evolution relative to this plan are evaluated by the Treatment Board^1^ to monitor the individual’s prison life and corresponding decisions.

## Materials and Methods

This study was conducted within the framework of the Spanish National Research Plan, “Processes of socio-educational reintegration and mentoring of inmates on temporary release”/“Procesos de reinserción socioeducativa y acompañamiento a reclusas en semilibertad,” REINAC (Ref.EDU2016-79322-R). The study was supported and approved by the Secretariat-General of Prisons and the Council for Justice of the Generalitat de Cataluña (Catalan regional goverment, the only entity with legal power in matters regarding the region’s prison system). The project studied 31 prisons.

### Goal

This study analyses early dropout, taking as reference the age at which basic levels of education are completed in the educational system (up to 18), the different risk factors influencing education, and the impact of prison on personal, social and job autonomy of women on temporary release in Spain. It also analyses the influence of these factors on social reintegration.

### Methods and Instruments

The study used qualitative and quantitative methods, applying them complementarily to the 310 mixed questionnaires and the 67 semi-structured interviews, designed *ad hoc* at a single point in time.

### Participants and Sample Design

The participating population consisted entirely of women in the second or third special level of the process of temporary release within the open environment of the Spanish prison system. The general sample was composed of 310 women inmates (30.1% of the total female population) in 31 prisons (5 in collaborating entities and 26 in the prison system).

The prisons represent the different resources available for serving one’s sentence in an open mode. These are termed Social Integration Centres, Open Sections, Outside Units for Mothers and entities collaborating with the prison environment. They are distributed across 13 of the 17 regions of Spain (Andalusia, Aragón, Principality of Asturias, Balearic Islands, Canary Islands, Castile and León, Catalonia, the Valencian Community, Extremadura, Galicia, the Community of Madrid, Murcia and the Basque Country).

To obtain the sample, we followed two-phase sampling:

(1) Selection of prisons according to regional representation and highest ratio of women.
(2) Once we entered the centres selected, random sampling criteria from among the women who wanted to participate and who fulfilled the requirement of having been imprisoned under the ordinary prison regime prior to temporary release.

The margin of error for the data (confidence level 95%) is 4.5 points.

### Procedure

This research was approved by the Ethics Committee of the General Vice Presidency for Institutional Relations and Regional Coordination, the Secretariat-General of Prisons, and the Council of Justice of the Generalitat de Catalonia, and governed by the University of Granada’s ethical principles for studies and research with human subjects.

The procedure for obtaining access to the participants was developed in coordination with each participating prison, which defined the different forms of contact based on the cases:

– In prisons with resident inmate population, the women were assembled in a specific place during times that the prison’s team deemed appropriate and that did not interfere with activities.
– If the women were working or had activities outside the prison, they were visited after they returned to the prison or before leaving it.
– The women who lived entirely outside the prison (serving their sentence by distance or on conditional release) and who returned for a regular appointment every 15 days or once a month (terms differed for different individuals) were phoned individually to request their participation and, if possible, to advance their regular appointment. In this way, we were able to create groups to facilitate the technical fieldwork by consolidating the team’s visits on specific days.

In all cases, we obtained voluntary formal written consent from all women in the sample.

The questionnaires were administered in person or in small groups and could be completed by the inmates themselves, with either full guidance (for participants who did not speak enough Spanish – 9.7% of the sample – or who had only rudimentary reading ability and/or comprehension) or partial guidance (completed by the individual or with help) as needed. We recognise that self-report bias can occur in self-completed questionnaires. To reduce the possibility of this bias, the authors implemented the study with a qualitative approach through interviews. Further, self-report is common practice in the literature, as it enables the extrapolation of results by observing consistency among the different studies that work with these variables and in future research (Moret-Tatay et al., 2016). The interviews were performed in isolated places directly between the participant and the interviewer, taking care that no prison personnel were present. The interviews were audio-recorded and lasted approximately 30–40 minutes. As the women interviewed had already completed the questionnaire, the interview focused on specific questions about their experiences prior to and during their life in prison, the consequences of prison and their current situation.

### Measurements – Data Analysis

The study analysed 308 questionnaires, completed by women who were old enough to remain in the education system (no university education, up to age 18). We created an SPSS database, and responses to the qualitative questions were transcribed and classified for subsequent content analysis. Based on these analyses, we distinguished the following subgroups:

(1) Women who had left the education system as minors (before the age of 18).
(2) Women who had stayed in or dropped out of the system as adults (18 years or older).

We applied descriptive statistics methods and independent contrasts to analyse the association of significant variables with school dropout, obtaining a log-linear model that related dropout to recidivism and Roma culture.

The average age of the women in the sample was 42.19 years, with a S.D. of 10.7. By country of origin, 71.8% were Spanish and 28.2% foreign, with 76.5% holding Spanish citizenship at the time of the study.

The questionnaire posed some questions that enabled us, after examination of the data and the personal interview, to establish subpopulations of “women who dropped out of school as minors,” a subpopulation totalling 205 women (66.6%); and “women who dropped out of school as adults,” 103 women (33.4%). Since only two women did not answer these questions, the final sample analysed in the study was composed of 308 women.

The subpopulations studied had some common characteristics at the time the questionnaire was administered, enabling comparison in terms of consistent educational and family data. The women had similar average ages, marital status and family situations (see data in Table 1).

**TABLE 1:** General sociodemographic traits in the two subpopulations.

Dropout		
	Minor	Adult
Age (average)	41.81 years (S.D. 10.523)	43.01 years (S.D. 11.078)
Marital status: partner	75 (37%)	31 (30%)
Marital status: no partner	128 (63%)	72 (70%)
Stable relationship	138 (67.3%)	60 (58.3%)
Children	182 (88.8%)	75 (72.8%)

*Source: the authors.*

As to jobs, in addition to the methodology used in other procedures, we analysed the changes in the women’s job status using the McNemar test to confirm significant changes between their previous job status and status at the time of the study. The factors with a positive influence on finding a job were classified using cluster analysis.

## Results

### Education Level and Variables Associated With Dropout

Table 2 shows a description of the data on education level, divided according to age at which the person dropped out of the education system and whether or not she had completed a specific level of education.

**TABLE 2:** Education level before prison in the two subpopulations.

	Minor			Adult		
Education/Dropout age	Total	Incomplete	Complete	Total	Incomplete	Complete
No education	34 (16.6%)	34 (16.6%)	–	–	–	–
Primary	101 (49.3%)	57 (27.8%)	44 (21.5%)	2 (1.9%)	2 (1.9%)	–
Secondary (ESO/BUP)	45 (22%)	29 (14.1%)	16 (7.9%)	23 (22.3%)	12 (11.7%)	11 (10.6%)
Vocational training (FP, official non-university	12 (5.9%)	7 (3.4%)	5 (2.5%)	17 (16.5%)	6 (5.8%)	12 (10.7%)
Non-compulsory secondary, COU –university-preparatory	13 (6.3%)	8 (4%)	5 (2.3%)	26 (25.2%)	7 (6.8%)	19 (18.4%)
Higher education	–	–	–	35 (34%)	8 (7.8%)	26 (26.2%)
Total	205 (100%)	135 (65.9%)	70 (34.1%)	103 (100%)	35 (34%)	68 (66%)

*Source: the authors.*

The results show that the women’s education level is matter for concern – and of even greater concern for the first subgroup of young women, who left the system as minors or did not attend school directly (16.6%). More women pursued primary education (49.3%) and secondary education (22%), although in both cases did not complete it. The reasons the participants in the 67 interviews gave for dropping out indicate predominantly economic problems in the family and the need to work, followed by situations that required them to help care for siblings and do house work, having a boyfriend and/or getting married, and, to a lesser extent, not wanting to study or not understanding the material taught.

This situation changes in the second subgroup of young women, who stayed in school for a longer portion of non-compulsory education (vocational training 16.5%, college-preparatory 25.2%), entering higher education in 34% of the cases, with the majority earning their degrees.

To define the profile of women who dropped out as minors or adults, we analysed the factors and consequences significantly related to dropout age in the subpopulations studied. Table 3 displays the percentages of women in each of the variables analysed. The percentages were compared using Chi-square independence contrast, obtaining *p*-values displayed in the fourth column. In all cases, these proportions differ significantly and are higher in the first subpopulation. The last column shows the odds-ratio, indicating for the variable analysed the ratio of higher incidence of this factor in the first subgroup than in the second.

**TABLE 3:** Factors related to dropout in the two subpopulations.

Dropout					
	Total	Minor	Adult	*p-value*	*Odds-ratio*
Relationship to Roma culture	130 (42.2%)	110 (53.7%)	20 (19.4%)	8.071*10^–9^	4.88
Criminal record	95 (30.8%)	76 (37.1%)	19 (18.4%)	0.001	2.63
Family members in prison	138 (44.8%)	114 (56.7%)	24 (23.3%)	7.1345*10^–8^	4.31
Children	257 (83.4%)	182 (88.8%)	75 (72.8%)	0.000376	2.95
Recidivism	75 (24%)	65 (31.7%)	10 (9.7%)	0.000022	4.32
Primary or secondary education in prison	176 (44.4%)	136 (66.3%)	40 (38.8%)	0.000023	3.14
Unemployment	114 (37%)	89 (43.4%)	25 (24.2%)	0.008	2.45
No job contract	63 (32.8%)	50 (40.2%)	13 (16.8%)	0.000174	3.78

*Source: the authors.*

Throughout the study, we provide detailed analysis of each of these factors and its relationship to dropout. Some of the general traits are:

#### Belonging to Roma Culture

42.2% of the women belonged to Roma culture. This percentage was 10 points higher in the first subgroup of women. The proportion of women leaving school as minors is 4.88 times higher if they belong to Roma culture. This risk factor thus has a very strong relationship to school dropout.

#### Criminal Record

30.8% of the women had a criminal record before entering prison. This percentage is 7 points higher in the first subgroup.

#### Family Members in Prison

44.8% of the women had or had had family members in prison, although these percentages differed greatly in the subgroups considered. In the first subgroup, 56.7% of the women had or had had family members in prison vs. 23.3% in the second subgroup. When both groups were asked which family members were in prison, the most common responses were “more than one family member” (40.4% of cases) and “partner” (29.2% of cases) for the first and second subgroups, respectively.

#### Children

83.4% of the women had children. Comparison of the percentages of the two groups shows a higher percentage for the first group, which also had on average more children (2.98 vs. 2.16). Overall, 40% of the women had adult children. As women were on average in their 40s, this means that they were young mothers, a fact that could have influenced why they dropped out of school early.

#### Recidivism

24% of the participants were recidivists. If we compare the percentages in the two groups, the risk that women return to prison is 3.26 times greater for women who dropped out of school as minors. These women had also committed more prior offences before entering prison and served more sentences.

The following sections describe the other main results presented in Table 3, complementing the analysis with other tests and data.

### Life in Prison: Education, Employment and Autonomy

#### School Education in Prison

Table 4 shows the formal education provided within the prison system.

**TABLE 4:** Education level: Formal education pursued in prison and during temporary release at time of study for the two subpopulations.

	Education in prison				Current education			
	Minors		Adults		Minors		Adults	
	N	%	N	%	N	%	N	%
Primary	86	61	9	25	25	62.5	1	1.1
Secondary (ESO)	37	26.2	7	19.4	8	20.0	2	2.2
Non-compulsory secondary	6	4.3	3	8.3	3	7.5	1	1.1
Higher education	4	2.8	7	19.4	1	2.5	5	5.6
Official language school	8	5.7	10	27.8	3	7.5	0	0
Total	141	100.0	36	100.0	40	100.0	9	100.0

*Source: the authors.*

While serving the sentence, 57.1% of the women (177) received education in prison (during their ordinary sentences) or were receiving it when they were on temporary release at the time of the study. While they were in prison, the first subgroup of women stressed that they pursued basic levels of education (61% primary and 25.7% secondary, or ESA/“Secondary Adult Education” since they were over 18). The second subgroup pursued higher education or language study at the official language school.

In the interviews, the number of women in the subgroup who dropped out of school as adults and answered that they had pursued these levels of compulsory education (primary and secondary) gave as their reason the need to occupy their time or to return to or update forgotten knowledge. In some cases, due to the limited educational opportunities provided, they had to repeat stages of education they had already passed. Although this schooling was not formally recorded, the women attested that they had taken these courses.

Next, we analyse the results concerning the women’s work situation prior to and while they were serving the sentence.

#### Unemployment

37% of the women were unemployed before entering prison. This percentage is nearly double in the first subgroup. Among the working women, the largest number worked in the hotel sector, the second-largest part of the first subgroup in cleaning and the second subgroup in caring for people.

#### No Job Contract

32.8% of the women who had worked before entering prison had no job contract, and this percentage was higher in the first subgroup of women. In this case, it is striking that 76.6% of the women with more education who had been working before entering prison had contracts, evidence of a clear difference in the two groups’ job stability. The differences in economic income were also considerable, as Table 5 shows.

**TABLE 5:** Economic income in the two subpopulations.

	Dropout/Economic income			
	Minors		Majors	
	N	%	N	%
Under 1000 euros	144	70.2	49	47.6
1000 – 2000 euros	42	20.4	34	33
2000 – 3000 euros	7	3.4	12	11.6
Over 3000 euros	3	1.5	5	4.9
Legally	8	3.9	3	2.9
DK/NA	1	0.5	0	0
Total	205	100.0	103	100.0

*Source: the authors.*

The women’s job status at the time of the study was different; the percentage of unemployed women had grown to 60.4%, yet only 10.1% had no contract; that is, the women had less work, but those with jobs worked in better conditions.

Analysing the change in the job situation (see Table 6) for each group separately, we obtain the following conclusions:

**TABLE 6:** Working or not working prior to prison and currently in temporary release for the two subpopulations.

Were you working before you went to prison?	Minors			Adults				
	
	No	Yes	NA	Total	No	Yes	NA	Total
No	61 (29.8%)	28 (13.7%)	0	89 (43.4%)	20 (19.6%)	5 (4.9%)	0	25 (24.3%)
Yes	61 (29.8)	49 (23.9%)	3 (1.5%)	113 (55.1%)	41 (40.2%)	36 (35.3%)	0	77 (74.8%)
NA	2 (1%)	0	1 (0.5%)	3 (1.5%)	0	0	1 (0.9%)	1 (0.9%)
TOTAL	124 (60.5%)	77 (37.6%)	4 (2%)	205 (100%)	61 (59.3%)	41 (39.8%)	1 (0.9%)	103 (100%)

	**Minors**			**Adults**				
	
	**Value**	**Df**	**Asympt.signif. (bilateral)**	**Value**	**Df**	**Asympt.signif. (bilateral)**		

McNemar-Bowker Test	17.236	3	0.001	4.59	–	<0.001		
N valid cases	205			103				

*Source: the authors.*

The marginal distributions show clear change. The McNemar test is significant and the *p-value* = 0.001 (minors), confirming the change in trend. Of these women, 29.8% had lost their jobs, while 13.7% had found work and 23.9% had maintained their jobs. Those who stayed in school until they were adults showed considerable changes in job status (*p-value* < 0.001): 39.8% of the women lost their jobs, only 4.9% found jobs and 35.3% kept their jobs.

Examination of the two groups shows that the percentage of women who dropped out of school as adults and lost their jobs is greater among women who dropped out of school as adults. These percentages are reversed, however, when it comes to finding a job. This phenomenon can be explained in this case by the fact that the women who dropped out of school as minors received more job training (69.3 vs. 52.4%), that is, were more predisposed to education than the women with a higher education level. In other words, women with higher education levels have more difficulty integrating into the job market after prison.

Table 7 shows that having taken a job training course influenced whether the women in both groups had jobs at the time of the study. Significant differences emerge within the total sample of women. Of the women who were employed at the time of the study, 68.4% had taken training courses, and 31.6% had not. In the individual analysis of the groups, we see that this difference is even greater – and statistically significant (*p-value* = 0.02) – for the women who dropped out of school as minors. In this group, the percentage of women who had jobs at the time of the study and had received training was 78.9%, as opposed to 48.8% who had not.

**TABLE 7:** Training for employment in prison and current job.

			Minor		Adult		Total	
	
Dropout age before entering prison			Current job Yes	*p*-value	Current job Yes	*p*-value	Current job Yes	Total *p*-value
Had taken classes or received job training	**No**	N	16	0.02	21	0.547	37	0.151
		%	21.1%		51.2%		31.6%	
	**Yes**	N	60		20		80	
		%	78.9%		48.8%		68.4%	
Total		N	76		41		117	
		%	100.0%		100.0%		100.0%	

*Source: the authors.*

We used cluster analysis to define the profile of the women currently working. The results show that the women who were working before they entered prison received vocational training courses, were not recidivists and had not dropped out of school as minors. Those who did not have jobs, in contrast, belonged to Roma culture, were recidivists and had family members in prison.

Detailed analysis of the relationship between the factors of belonging to Roma culture and being a recidivist in the total group of women show that this relationship is significant. On considering the groups based on dropout age, however, we find a real significant association among these factors for women who dropped out as minors but not for those who remained in school until the age of 18. Nearly three times the number of women who were recidivists belonged to Roma culture (73.8 vs. 26.2%). The inverse relation holds, however, if the women ceased their education when adults, as only 10% of these women were of Roma culture. To contrast whether the analysis of these variables is accurate, we considered the multiple combinations of log-linear models that can be obtained with these three variables. The best-adapted model was the one with the lowest Akaike Information Criterion (AIC).

Table 8 shows the results of comparing the different models obtained, identifying the variables with codes for simplicity:

**TABLE 8:** Log-linear models applicable to relationship structure among the 3 variables.

Model	GL	Statistic	*p*-value	AIC
[12][13][23] Pairwise independence	1	4.768	0.029	2.768
[12][13] Conditional independence	2	15.1	0.001	11.1
[12][23] Conditional independence	2	15.555	<0.0001	11.555
[13][23] Conditional independence	2	30.606	<0.0001	26.606
[3][12] Partial independence	3	35.252	<0.0001	29.252
[2][13] Partial independence	3	50.303	<0.0001	44.303
[1][23] Partial independence	3	50.758	<0.0001	44.758
[1][2][3] Global independence	4	70.455	<0.0001	62.455

*1: Dropout age. 2: Relationship to Roma culture. 3: Recidivism profile. Source: the authors.*

The best model has as generative class [12][13][23] the model of partial association, or model of absence of interaction, among the three factors. That is, there is a significant relationship between each pair of variables, as we concluded from the individual pairwise analyses.

### Autonomy for Reintegration and Facing Life in Freedom

In the preceding section, we described the women’s situation concerning schooling, job training and having or not having a job at this advanced stage of their sentence – temporary release. We must also consider other elements, however, elements related to attitudes and expectations concerning full freedom.

Among these factors, maintaining our focus on capability for reintegration in the job force, we asked the women about their attitudes toward employment (see Table 9). The items that showed significant differences (20–40 points of difference between the populations) were knowing how to prepare a CV for a specific job, handling a job interview and using a computer, internet or mobile devices.

**TABLE 9:** Skills and competences for current educational/work-related autonomy.

Attitude	Minor	Adult	*p-value*
1. I know how to write or adapt my CV for a specific job	129 (62.8%)	88 (85.5%)	<0.0001
2. I know how to handle a job interview	164 (79.8%)	95 (92.2%)	0.009
3. I feel prepared and trained to start working	185 (90.3%)	93 (90.3%)	0.87
4. I take orders at work	195 (95.1%)	95 (92.2%)	0.33
5. I respect work hours (starting time, breaks, leaving time)	195 (95.1%)	101 (98%)	0.28
6. I assume responsibilities	199 (97.1%)	100 (97%)	0.78
7. I work and cooperate on a team	193 (94.1%)	101 (98%)	0.17
8. I use a computer	91 (44.4%)	92 (89.3%)	<0.0001
9. I use internet	110 (53.6%)	95 (92.2%)	<0.0001
10. I use mobile devices	156 (76.1%)	98 (95.1%)	<0.0001

*Source: the authors.*

We also performed cluster analysis to identify which items (in Table 10) were more closely related in women who were working at the time of the study. This analysis shows that these women clustered around items 4, 5, 6, and 7. In last place was item 9, an attitude of believing they are less prepared.

**TABLE 10:** Facing freedom, and personal and social autonomy.

	Dropout			

	Minor		Adult	
	N	%	N	%
1. I am afraid of what I will find out there when I am finally released	65	31.7	37	36
2. It is hard for me to manage administrative matters (make doctor’s appointments, social services, employment services, etc.)	58	28.3	27	26.2
3. I feel insecure when I go out on the street and encounter new things	45	22	23	22.4
4. I feel insecure about making decisions	62	31.7	33	32.1
5. I need the help and encouragement of others to make decisions	68	33.2	34	33
6. I have accepted letting my family/others make decisions for me	60	29.3	28	27.2
7. I have a hard time performing basic activities of everyday life (cooking, hygiene, getting enough rest)	15	7.4	9	8.7
8. I cannot manage my own affairs (professionals and other people continue to do it for me)	41	20	15	14.5
9. I solve day-to-day problems, and it is hard for me to plan for the future because I don’t know what is going to happen	112	54.6	43	41.8
10. It is hard for me to adapt to work routines	14	6.9	18	17.5
11. When I am finally free, everything will go well	171	83.4	86	83.5

*Source: the authors.*

No significant differences emerge in the two subgroups of women for questions of facing life in freedom, with the exception of the question of adapting to work routines (*p-value* = 0.003). In general, these low percentages mean that the women adapt well to work routines, but the women with a higher education level from the second group show a higher percentage, more than double that of the first subgroup.

Note that the women with the lowest percentages had the greatest difficulty adapting to work routines and performing basic activities of everyday life. Those with the highest percentages were very optimistic, thought that everything would go very well and believed that they were capable of solving day-to-day problems (see Table 11).

**TABLE 11:** Self-evaluation of preparation for freedom.

	Minor		Adult	
	Frequency	%	Frequency	%
Very poor	3	1.5	3	2.9
Poor	4	2.0	3	2.9
Fair	35	17.1	15	14.6
Good	71	34.6	29	28.2
Very good	88	42.9	53	51.5
No answer	4	2.0	0	–
Total	205	100.0	103	100.0

*Source: the authors.*

Both groups evaluated their preparation for life in freedom as very good; 78.6% of the women rate this preparation as good or very good. The two things that the women indicate would help them to prepare for life in freedom were, first, “family support” (35.1%) and “having work” (26%), factors evaluated equally by both groups of women; and second, for the women who dropped out of school as minors, the choices “love of my family” (17.1%) and “having a job” (13.7%); and for the women who dropped out as adults, the choices “having a job” (13.6%) and “love of family” (11.7%).

The participants also evaluated the consequences that having been in prison would have for their lives. The items with the highest percentages of positive responses in women who dropped out of school as minors were related to situations not experienced in freedom; the sound of the bars (67.5%), the sound of the loudspeakers (66%) and the anxiety caused by gatherings (60.5%). Among the second subgroup of women, the most significant items were also related to the sounds of prison (67 and 67.9%, respectively) and the fear or shame of having been in prison (62.2%). All of these factors correspond to the psychological pressure that these women feel at having been in prison, and they are frightened when they face life outside prison.

## Discussion

**FIGURE 1:**
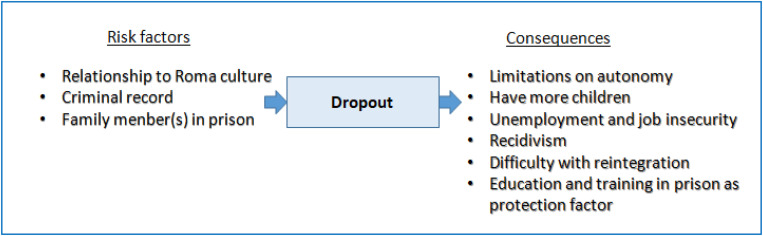
Variables related to early dropout. Source: The authors.

By examining the results of the variables significantly related to dropout when one is a minor or an adult, we can establish a risk-consequences relationship that enables us to understand the life trajectories of the women sentenced to prison from their childhood and youth until the time of the study (Figure 1).

We start by exploring the realities of prison. In addition to loss of freedom, these realities are shaped predominantly and in themselves by histories prior to the sentences, realities permeated by disadvantaged personal, social, economic, educational and other contexts (Añaños-Bedriñana, 2013). The rate of early dropout in the women studied is a very important indicator or factor (66.6%) relative to the general Spanish population: 14.0% in 2018, the year of the study (INE, 2018). We also see that minors have a lower level of primary education (no or incomplete education) in 29.6% of the cases, while 13.3% did not complete secondary education. Of the other group of adults, in contrast, 29.2% pursued and completed secondary study, vocational training or higher education. One’s career record in the education system has historically been considered a fundamental indicator of the subsequent path of adolescents and young people (Nieto, 2011). These data confirm the force of its importance. A low or medium-level educational profile reveals the urgent need to strengthen adherence to basic education levels and to foster and protect the right to education in the prison environment (Añaños et al., 2019).

The results show, first, that *Roma culture* is one of the most prevalent traits in early dropout (4.88 times higher than non-Roma participants). Our sample contained 130 Roma women (42.2%). In this subgroup, 110 (85%) had left school as minors and only 20 (15%) had completed basic education. Belonging to this ethnic group coincides with minimal or no attendance of compulsory levels of education. Even outside prison, Roma show high percentages of absenteeism and early dropout – up to 96%. Only 17% of the Roma population over 16 has completed ESO or higher education levels (De la Rica et al., 2019).

If we also consider gender, we see that these Roma women suffer from fourfold discrimination stemming from their social condition, their ethnicity, having been in prison and being women (Pérez de la Fuente, 2008; Añaños-Bedriñana, 2012). At the public level, being a woman is perceived as an important agent of social change, but women in the private sphere are conceived as the weakest and most oppressed party in Roma society (Ayala Rubio, 2014). Cárdenas-Rodríguez et al. (2019) find that absenteeism has been eradicated in primary education but that female Roma students usually have a higher dropout rate in subsequent stages (around 15–16 years of age), since families view this as the age at which to get married, care for the family or household, and reduce likelihood of a future with low expectations for education. Spain’s National Roma Integration Strategy 2012–2020 aims to strengthen completion of compulsory education among the Roma, especially women.

Although the Roma ethnic group has advanced significantly, it continues to be one of the sectors with the greatest difficulty participating in society (Laparra and García, 2011). Roma are Spain’s main indigenous minority (Gamella, 2011), and they continue to experience processes of structural exclusion (Latorre-Arteaga et al., 2017). All of these factors influence various personal and social dimensions. No official data exist on the Roma ethnic group in the prison environment. Collecting such data would be unconstitutional, as it exposes or makes visible a discriminatory conception of ethnicity (Article 14 of the Spanish Constitution). Based on the Map of Housing and Roma Community (Ministerio de Sanidad, Seguridad Social e Igualdad [MSSSI], 2012), we estimate a population of approximately 516,862 (Fundación Secretariado Gitano, 2016). When related to this study, in which Roma women represent 42.2% (130 women), this figure shows a disproportionately high presence of Roma women in the prison system. The Asociación Pro Derechos Humanos de Andalucía (2020) also indicates a high number of women of Roma ethnicity in prison in Spain, 25% in 2005.

*Having a family member in prison* (44.8%) is another risk factor (Nieto, 2011) in 56.7% of minors and 23.3% of adults. We infer that the environment surrounding the person, especially the young person, has a significant influence on disposition to commit crimes, and that close family members (siblings, parents) and partners are especially influential. The data are similar for *criminal record* (30.8%). Of the women who had committed formally reported crimes prior to the current sentence, 37.1% were from the first group and 18.4% from the second. Later we will detail how these situations relate to the consequences of dropout, resulting in 24% *recidivism* (31.7% in minors, 9.7% in adults).

Early dropout, not having a degree or failing in school have been defined in the literature as possible risk factors for crime (Aaltonen et al., 2011; Bäckman and Nilsson, 2011). Studies such as Sweeten et al. (2009) establish that individuals who have dropped out of secondary school are more likely to commit crimes (Deming, 2011). They also establish that quality of education can be a predictor of subsequent criminal behaviour. Anderson (2012) proposes that school can encourage one to occupy one’s time and thus reduce opportunities to commit criminal acts, confirming the importance of the education process as a tool for preventing risk behaviour.

Another consequence is *young motherhood*, which is more frequent in women and girls who drop out of school (88.8 vs. 72.8%), as is having a larger number of children (2.98 vs. 2.16) and having independent children (40% of the women). The average age of the women studied (40 years old) suggests that they became mothers very young, a factor that may have influenced early dropout. Motherhood involves adaptation to a new situation that requires new strategies, as social condition is a significant determining factor (Mercer, 2004). Roma woman are prepared for this process (Fernández Morate, 2000), since they are educated culturally to become mothers and wives (Márquez and Padua, 2009; Montanñés Álvarez, 2011) at a very young age, but the difficulty of succeeding in the education system and socially grows significantly.

Dropping out of school plays a determining role in *conditions for entering the labour market* (Fortin et al., 2005; Eckert, 2006). First, the women have fewer skills and attitudes that are advantageous for re-entering job market, with differences between the two subpopulations (20–40 points) and higher levels of recidivism (3.26 greater than those who continued school until the age of 18). Second, being unemployed, having job insecurity or not having a work contract are intimately related to fragility of both the social and the educational support (Salvá, 2009) that motivate one to perform other kinds of activities as a means of subsistence. In the study, 37% of the total sample and 43.3% of those who dropped out as minors were unemployed before entering prison, and of the women who had jobs, 32.8% had no contracts. Of those who did have contracts, however, 76.6% belonged to the second group. Unemployment has currently grown to 60.4%. Only 3.9% of the women had jobs both before entering prison and at the time of the study, and of those working, only 10.1% had no contract. These data confirm a change in labour trends, but precarity, early disadvantages and/or exclusion can intensify these trends (Fortin et al., 2005; Añaños-Bedriñana, 2012, 2013; Esteban et al., 2014), adding the risk of crime and further increasing social stigma.

Socioeconomic level combined with a lower cultural level can thus result in low school performance (Ruiz, 2001; Vera et al., 2005). The reasons the women in the study mentioned for dropping out of school were fundamentally socioeconomic, explained by the minimal importance school had for them. School did not generate immediate wealth, whereas criminal activity yields greater income more quickly to help the family and to help them meet their expenses (Henríquez, 2017). We see that 70.2% of minors vs. 47.6% of adults earned a salary under 1000€ in the entire family unit, an income insufficient to support quality of life that satisfies all basic needs.

Once in prison, the women’s *educational careers changed*. We find 57% who had received formal education. According to their self-perceptions, this education provided them with more entertainment, occupied their minds and helped them to recall learning received in the past. The positive side of this situation is that it signified an enriching use of time and provided emotional well-being (García-Vita and Melendro Estefanía, 2013; Monteserín and Galán Casado, 2013; Añaños-Bedriñana and Garciá-Vita, 2017). This approach mitigates the perspective of the prison environment and its routines, which infantilizes and institutionalizes, since socio-educational intervention and daily activities can encourage association with autonomy and well-being (Goodstein et al., 1984; Gover et al., 2000; Liebling, 2004; García-Vita and Melendro Estefanía, 2013; Van der Laan and Eichelsheim, 2013). These effects in turn give inmates more structure to their lives in prison and positive interactions with prison personnel and fellow inmates, producing positive effects once the legal punishment ends (Van der Laan and Eichelsheim, 2013). Education also provides quality of life during the inmates’ lives together in the prison environment.

This line of argument illustrates the *difficulty of inclusion, development and exercise of autonomy*, as well as deprivation of freedom. First, those who drop out as minors have more limited skills and competencies to face searching for and performing a job (knowing how to adapt one’s CV, performing an interview and especially handling ICTs – computers (55.6%), internet (46.4%) and mobile devices (23.9%). Second, at the personal level, difficulties arise in managing and facing freedom, difficulties related to solving everyday problems and planning for the future (46.4%), fear of uncertainty about what will happen (31.7%) and limitations in personal self-management (20%). We do not find differences in the two subgroups’ insecurity in decision-making; family and other people intervene in their decisions, and they show greater dependence on other people and professionals, indicating difficulties not only with personal autonomy but also with relational autonomy and interaction of choices, skills and resources (Ennuyer, 2002; Le Coadic, 2006). Despite these data, 78.6% of the two subpopulations evaluated themselves very positively concerning their preparation for and facing of freedom, and the belief that life in freedom would go well (83.5%). These self-perceived strengths indicate their preparedness to start a job, take orders at work, respect schedules, assume responsibilities and work on a team. Such qualities show some beneficial effects to mitigate the destabilizing effect of the prison environment (Wormith, 1984; Zamble and Porporino, 1988; Zamble, 1992; Johnson and Dobrzanska, 2005).

We thus find somewhat contradictory perspectives on these women’s realities and initial situations, and the expectations and circumstances they face in their current everyday lives. It is clear that the prison environment does not encourage a normalized lifestyle, that it affects – among other issues – decision-making and the free development of autonomy, since inmates are conditioned to the established routines and itineraries as part of life in prison (Johnson and Dobrzanska, 2005), as well as to multiple rules and controls for security and constraints on living together. In these conditions, the better one fits into or adapts to the prison culture, the less one develops autonomy (Goodstein, 1979).

The women studied evaluate the job training they obtained in prison positively. Of the women who had jobs at the time of the study, 68.4% had taken these courses, and this figure increases in the case of the women who dropped out of school as minors (78.9%). The main characteristics related to job status were whether the women had jobs at the time of the study, had jobs before entering prison, had taken vocational training courses in prison, had not been recidivists in crime and did not drop out of school as minors. Those who did not have jobs either belonged to Roma culture, had a recidivist profile or had family members in prison. These findings show that participation in such training programs has given the women more opportunities to find work after prison, even though they have a lower education level. We must clarify, however, that the jobs they find are low-skilled and precarious. Precarity can lead to job dissatisfaction (Roulstone and Warren, 2006), raising further interconnected personal, emotional and social problems.

Job training activity – whether delivered in treatment programmes (educational, job training or specific programmes) typically located in the school and/or sociocultural spaces or in the workplace itself – can be understood as a factor that encourages autonomy (Parrón, 2014) and helps the women to acquire strategies for a more normal life adapted to the context they will enter in freedom. Thus, education is a fundamental support enabling the person to achieve greater control of his/her life, both in prison to avoid or minimize behaviours such as prisonization (Goodstein, 1979) and outside prison (Johnson, 2002). To these factors, we must add the formation of values, networks, attitudes, competencies, projections, etc. that encourage social reintegration.

The evidence obtained indicates that dropout or failure occurs due to the influence of individual, social, economic and cultural factors that combine simultaneously (Rumberger and Lim, 2008; Espínola and Claro, 2010). In all cases, the exposure and vulnerability of individuals who cease their education as minors is greater than that of those who stay in school longer. Many of these women surely return or will return to their initial environments and the situations described above after their sentences, and these environments are risk factors if the women have not previously undertaken psychoeducational and social work for real social integration-reintegration and prevention of recidivism. Further, society labels these women, stigmatizes and excludes them (Migallón and Voria, 2007; Añaños-Bedriñana, 2012, 2013), influencing or limiting their development toward assuming a sense of belonging to the community (Western et al., 2015), of which they are still a part.

In sum, educational activity, combined with social support, mentoring and socio-job-related counselling (Laub and Sampson, 2001; Sampson and Laub, 1993; Petersilia, 2003), as well as various social and community resources, can encourage processes of successful transition, leading to reduction of vulnerabilities and improvement of individual and social opportunities (Caride and Gradaille, 2013). Actions taken from multiple professional perspectives in the prison environment, among which we stress socio-educational action, should thus be oriented to developing autonomy and empowerment, recognizing these qualities as agents of change (Valderrama, 2013; Behan, 2014; Añaños-Bedriñana and Garciá-Vita, 2017), and awakening social consciousness and responsibility (Rotman, 1986) so that inmates can decide for themselves and participate critically in society (Martín et al., 2013). These premises agree with other studies that show that engaging in such processes enables inmates to improve their personal condition while also giving meaning to the sentence imposed (Viedma, 2013; Vázquez Cano, 2013), making these processes a factor for preventing delinquency (Sweeten et al., 2009; Aaltonen et al., 2011; Bäckman and Nilsson, 2011; Deming, 2011). Such work is not easy. As mentioned at the start of this study, the very nature of prison as an institution and the impact of its subculture on inmates interferes with efforts to empower them for the free exercise of autonomy (Clemmer, 1958; Goodstein, 1979; Goffman, 1987).

The school, personal and social paths analysed significantly limit the trajectories of these women’s lives and directly impact their autonomy and capability for participation as citizens in a demanding society in constant change. This situation calls on us to address the challenge of proposing educational, social, psychological and other types of processes that, combined with quality education suited to people’s real situation, encourage these women to achieve factors that protect against and reduce or counter the risk factors. All of these conclusions directly imply greater autonomy for inclusion and social integration-reintegration under better conditions and opportunities, conditions that go beyond mere satisfaction of needs for material autonomy (work, housing, food, resources, follow-up support for the women’s social reintegration, etc.). Further, these premises enable individuals to grow by developing themselves in a liberating process of emancipation as human beings, as individuals who create their own sustainable, autonomous path in life (Añaños-Bedriñana, 2013; Huber, 2016). And education can help them to fulfil this process.

## Data Availability Statement

The raw data supporting the conclusions of this article will be made available by the authors, without undue reservation.

## Ethics Statement

The studies involving human participants were reviewed and approved by Comité Ético de la SGIP and Consejería de Justicia de la Generalitat de Cataluña. The patients/participants provided their written informed consent to participate in this study.

## Author Contributions

FA: theoretical framework, discussion, and PI of the project. MG-V: theoretical framework, discussion, and interpretation of the results. DG-C: theoretical framework and discussion. RR-M: statistical analysis of the data and interpretation of the results.

## Conflict of Interest

The authors declare that the research was conducted in the absence of any commercial or financial relationships that could be construed as a potential conflict of interest.

## Footnotes

1 Treatment Board: A professional entity composed jointly of the technical team and the prison’s leadership to evaluate the case of each inmate.
